# The mediating role of cardiac patients’ perception of nursing care on the relationship between kinesiophobia, anxiety and depression in rural hospitals: a cross-sectional study

**DOI:** 10.1186/s12912-024-01875-3

**Published:** 2024-04-10

**Authors:** Mohamed Hussein Ramadan Atta, Shimmaa Mohamed Elsayed, Sharaf Omar Al Shurafi, Rasha Salah Eweida

**Affiliations:** 1 Psychiatric and Mental Health Nursing Department, Faculty of Nursing, Alexandria University, Alexandria City, Egypt; 2 Lecturer of Critical Care and Emergency Nursing, Faculty of Nursing, Damnhour University, Damnhour City, Egypt; 3 Al Aqsa University, Gaza City, Palestine; 4 Psychiatric and Mental Health Specialty, Nursing Department, College of Health and Sport Sciences, University of Bahrain, Manama City, Bahrain

**Keywords:** Cardiac patients, Anxiety and depression, Kinesiophobia perception of nursing care, Rural hospitals

## Abstract

**Background:**

Kinesiophobia could act as a significant barrier against physical activity following cardiac procedures worsening cardiovascular health problems and potentially leading to conditions like hospital-acquired anxiety and depression among patients with cardiovascular disease (CVD). Nurses are the vanguard health care team who can aid patients in taking proactive steps to overcome fear of movement following cardiac procedures.

**Aim:**

The overarching aim is to investigate the relationship between kinesiophobia, anxiety and depression, and patients’ perception of nursing care.

**Method:**

A descriptive correlational research design in two rural hospitals, conducted at cardiac intensive care units of Kafr Eldawar Hospital and Damanhur Medical National Institute. Data were collected from 265 nurses, using the following patient-reported outcome measures, the Tampa Scale for Kinesiophobia (TSK), the Hospital Anxiety and Depression Scale (HADS), the Person-Centered Critical Care Nursing Questionnaire (PCCNP) and the patients’ demographic and clinical profile.

**Result:**

A significant negative correlation was found between HADS and PCCNP (r: -0.510, *p* < 0.001) however, Kinesiophobia was significantly and positively correlated (r: 0.271, *p* < 0.001). A direct effect of PCCNP in the presence of the mediator was found to be not statistically significant (-0.015, CR = 0.302, *p* = 0.763). Nonetheless, PCCNP indirectly affects kinesiophobia through HADS (*p*=-0.099).

**Implication for nursing practice:**

Customizing individualized cardiac rehabilitation (CR) programs based on the emotional experience of cardiac patients will be conducive to rehabilitation and prognosis for patients, thereby lessening the physical burden and improving their quality of life.

## Background

According to World Heart Federation statistics, 20.5 million people died from cardiovascular disorders (CVD) in 2021, while over 500 million individuals globally still struggle with these conditions as of 2023 [1, 2]. More specifically, 6 out of 21 nations in the Middle East and North Africa had higher than average death rates from cardiovascular disease. Egypt is among the Middle Eastern countries with a high incidence of cardiovascular mortality, in which 600.0 women and 491.6 men per 100,000 inhabitants died from CVD. According to the World Health Organization (WHO), the increased incidence of risk factors like obesity, hypertension, and diabetes is among the most prevalent determinants associated with the prevalence of heart-related illnesses in Egypt [2, 3].

The most recent guidelines from the European Society of Cardiology recommend the significance of exercise and physical activity (PA) in enhancing lifestyle and preventing cardiovascular disease (CVD) [4]. Nonetheless, the WHO projects that between 60% and 85% of the global population have sedentary lives, and inadequate physical activity accounts for over 3.5% of annual fatalities [5, 6]. Emerging evidence suggests that kinesiophobia, or fear of movement, could act as a significant barrier against physical activity following cardiac procedures. kinesiophobia may potentially hinder rehabilitation efforts and affect the willingness of post-CVD patients to engage in physical activity. Cardiac pain could lead to various negative psychological ramifications, such as increased restrictive behaviors [7]. Kinesiophobia is defined as “an extreme, illogical, and crippling fear of physical exercise and movement caused by a perception of vulnerability to painful injury” [8]. Bäck et al. noted that a significant proportion of cardiac patients experience high levels of kinesiophobia, with approximately 20% reporting this fear [9]. However, Nair et al. found that 86.7% of patients undergoing cardiac surgery procedures experienced preoperative kinesiophobia [10].

There is a devoid of information related to the causes of kinesiophobia or fear of movement in patients with CVD. It is most probably related to the experienced physical manifestations including shortness of breath, chest pain, or an increased chance of another cardiac episode [7, 11]. Unfortunately, avoiding physical activity can feed a vicious cycle of aggravating cardiac disease and raise the risk of cardiovascular complications by causing deconditioning, decreased cardiovascular fitness, and thereby undermining their overall quality of life [12–14].

Equally important, kinesiophobia can be exacerbated by co-occurring mental health problems such as sadness, anxiety and depression [15–18]. In more recent studies, mental health issues are quite prevalent in cardiac patients; estimated up to one-third of people with CVD are suffering from anxiety and depression [15, 19]. Bahall et al., reported that comorbid depression and anxiety have significant negative effects on patient’s health, which further discourages patients from engaging in physical activities. Paradoxically, management and rehabilitation of CVD depend heavily on regular exercise and physical activity. In this sense, we believe that addressing cardiac patients’ perception of nursing care would help to overcome feelings of kinesiophobia and other hospital acquired anxiety and depression [16].

Cardiac patients’ perception of nursing care can impact how open they are in receiving medical advice, and how they interact with healthcare providers including nurses [20]. Nurses are the vanguard health care team who ought to take a patient-centered approach and attend to both psychological and physical requirements [21]. They also play a crucial role in providing psychological care tailored to cardiac patients to manage pain, engage in physical activity, and prevent complications that may arise from inactivity [22, 23]. Nurses can help patients take more proactive steps to boost their stress tolerance and adaptive coping with illness. In this regard, if the patient positively appraises the nursing care accorded to him, he/ she would be able to curb feelings of fears and limits related to kinesiophobia as well as the associated feelings of emotional discomfort [7, 22, 24, 25].

Patients who suffer from depression and kinesiophobia frequently find it difficult to control their conditions. Indeed, improving clinical outcomes of cardiac patients can be greatly aided by the nursing care [22, 23, 26, 27] Nurses may lessen the obstacles caused by kinesiophobia and comorbid illnesses by giving patients compassionate, patient-centered care that makes patients feel heard, supported and understood. Besides, adopting competent nursing care to these situations can enable patients to actively participate in their care more, improving their quality of life and thereby their clinical outcomes [24–26].

Based on the findings from studies such as Wang et al. (2023) [31], three types of kinesiophobia were identified in patients with coronary heart disease: low fear, intermediate fear, and high fear. Keessen et al. (2022) [28] found that moderate and severe levels of kinesiophobia were associated with cardiac anxiety. Additionally, Yükselmiş Ö [29]observed that individuals with increased kinesiophobia experienced more anxiety/fear of falling and higher levels of depression. Ratnoo et al. (2023) [30] reported that patients following Coronary Artery Bypass Grafting exhibited moderate levels of anxiety and depression, along with a high level of kinesiophobia.

Regarding nursing care practice and kinesiophobia, Wang et al. (2023) [31], delineated the perceptions and practices of cardiac surgery nurses regarding kinesiophobia management. The study highlighted a scenario characterized by a high level of recognition but limited engagement among nurses, coupled with deficits in knowledge retention and a lack of willingness to address kinesiophobia. The authors underscored the necessity of advancing kinesiophobia management through the implementation of key strategies, including the adoption of an effective health education model, fostering stable collaboration between medical staff and family caregivers, streamlining clinical protocols, establishing specialized nursing teams, and delineating clear lines of multidisciplinary responsibilities. In addition, Bastani, et al. in 2022 [32], focused on examining how the quality of nursing care relates to anxiety and depression in patients with CVD. The findings from this research affirm the significant impact of care quality on anxiety and depression levels among patients with CVD.

To our knowledge, this is the first study examining the correlation between kinesiophobia, emotional state, and perception of nursing care among cardiac patients at both national and international levels. Therefore, this study provides fertile ground for mapping the factors correlated with kinesiophobia and how kinesiophobia impacts mental and physical health outcomes in patients with CVD. This would ultimately aid in adequate support for these patients as well as improving their functional capacity. Moreover, our work addresses a significant gap by calling for prioritizing this pressing issue on the nurses’ agenda, as they typically engage in direct patient’ care. Consequently, it can offer valuable insights into the clinical application perspective for the proper management of kinesiophobia. This study will consider perception of nursing care as a feasible mediator in the relationship between kinesiophobia, and anxiety and depression among cardiac patients in rural hospitals. Given the foregoing literature, we hypothesize that:

### Hypothesis 1

Perception of nursing care is negatively correlated with kinesiophobia.

### Hypothesis 2

Kinesiophobia is positively correlated with anxiety and depression.

### Hypothesis 3

Perception of nursing care plays a mediating role between kinesiophobia, anxiety and depression.

### Aim of the study

The overarching aim is to investigate the relationship between kinesiophobia, anxiety, and depression and patients’ perceptions of nursing care among patients with cardiovascular disease. Further objectives are to predict factors that affect feelings of anxiety, depression, and kinesiophobia and to analyze the mediation role of patients’ perceptions of nursing care on the associations between kinesiophobia, anxiety, and depression.

## Methodology and materials

### Design

A descriptive correlational research design was adopted.

### Setting

The research was conducted at the cardiac care units of two rural hospitals, namely Damanhur Hospital and Kafr Eldawar. Each hospital’s cardiac intensive care unit (ICU) has a total capacity of 50 beds.

### Ethics

The study protocol received approval from the Research Ethics Committee of the Faculty of Nursing, Damanhur University (**RES: 65-b**). Before their involvement in the study, all participants provided informed consent or appropriate representative (relative), with full knowledge that their participation was voluntary and they had the right to withdraw without facing any consequences. Throughout the study, strict measures were taken to ensure the confidentiality of the participants.

### **Participants** & sampling

The study employed systematic randomized techniques to select participants in the total number of cardiac patients. This data collection followed rigorous guidelines to ensure the validity and reliability of the study’s results.

The sample size was calculated using the G*Power Windows 3.1.9.7 program, with a power of 0.95, an effect size of 0.15, an alpha error probability of 0.05, and several predictors = 2. Using Based on the calculation, this study required an a priori sample size of 215 patients randomly, the researcher decided to recruit 270 patients after considering a 20% loss ratio of follow-up. The statistician tried to match the eligible criteria, to be eligible, participants were diagnosed as cardiac ill patients, did not have any musculoskeletal problems (e.g.; handicapped, osteoporosis) and were less than 60 years old to control for the covariates of osteoarthritis and depression-related medical conditions as a confounding factor [33].

To ensure that all eligible patients were properly represented in the study sample, the systematic randomization sampling technique was used through the steps mentioned. Initially, the list of patient’s names who were admitted was considered. Then, systematic randomization sampling was conducted based on the systematic rule of using a fixed interval. In the selection process, the researchers include the last patient from every 3 patients (i.e., 3, 6, 9, etc.). The total sample size consisted of 270 patients, including cardiac patients. Five of those patients refused to participate in the study. The final sample size was divided into an equal sample size (265) that was selected from Kafr Eldawar Hospital and Damanhur Medical National Institute (Fig. 1). Each randomly selected patient was screened to identify those who met the predetermined inclusion criteria. Steps were repeated until the number of decided-upon subjects was reached. Each recruited subject was interviewed individually to establish rapport and apply tool I, followed by II, III, and IV.

Each patient was interviewed individually to establish rapport and collect data related to the measured outcomes. The researchers provide patients with clear, structured, and standardized tools that are relevant to the topic being assessed to mitigate any potential biases and ensure objectivity throughout the data collection. Data collection was conducted over two months between the beginning of July 2023 and the end of August 2023.

### Flow graph

**Fig. 1:**
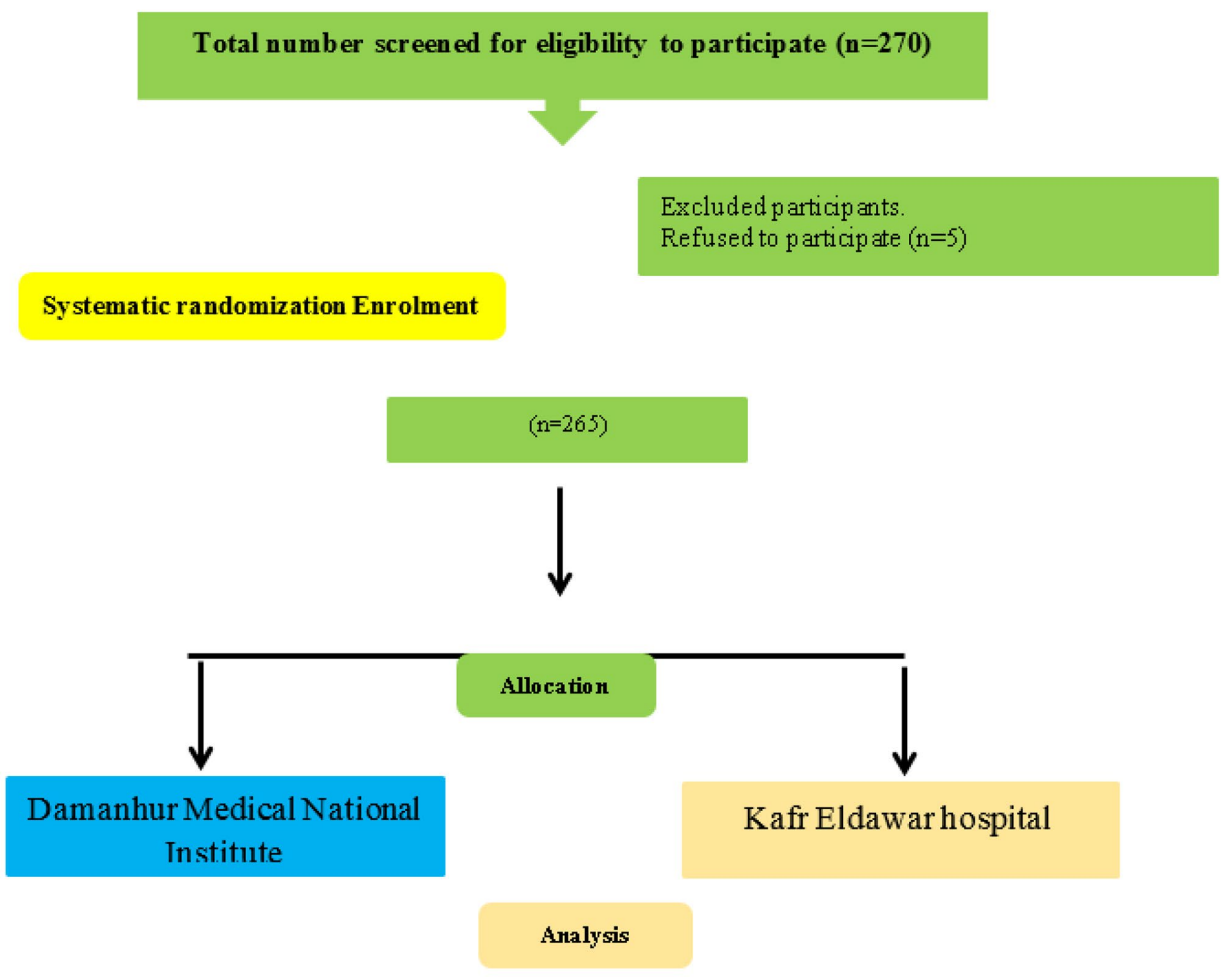
Flow graph

### Measured outcomes

This study employed four different instruments to gather data:

Tool I: a structured form related to the social and clinical profile of patients was divided into two parts: Part 1: collected socio-demographic data, such as the patient’s gender, age, place of residence, and marital status. Part 2: collected clinical data, including diagnosis period, history of illness.

### Tool II: The Tampa Scale for Kinesiophobia (TSK)

The TSK questionnaire, which was developed in 1991 by Miller R., and Kori S [34], is a tool used to measure fear of movement. It aims to assess a patient’s excessive, irrational, and debilitating fear of physical activity, which stems from a perceived vulnerability to painful injury. The questionnaire consists of 17 items, and respondents rate their agreement on a 4-point Likert scale ranging from “Strongly Disagree” to “Strongly Agree.” Total scores on the TSK range from 17 to 68, with lower scores indicating minimal or no fear of movement and higher scores indicating a greater degree of kinesiophobia. The TSK has been found to have strong internal consistency across all items [35]. The reliability of the Finnish version of the TSK, as measured by test-retest reliability, was found to be 0.887 [36]. In the current study, the Arabic translation of the TSK demonstrated good internal consistency, as indicated by Cronbach’s α value of 0.88.

### Tool III: Hospital Anxiety and Depression Scale (HADS)

The Hospital Anxiety and Depression Scale (HADS) is a questionnaire consisting of 14 items, with seven questions dedicated to measuring anxiety and seven questions for measuring depression. Each question is scored on a scale from zero (indicating no impairment) to three (indicating severe impairment), resulting in a maximum score of 21 for both anxiety and depression [37]. The HADS has been widely used to assess anxiety and depression in cardiac patients, and a study by Amin et al. (2022) [38] reported a Cronbach’s alpha value of 0.70, indicating good internal consistency. To aid in the interpretation of scores, a classification scheme can be applied: scores ranging from 0 to 7 suggest the absence of clinical symptoms, scores between 8 and 10 indicate moderate levels of depression or anxiety, and scores from 11 to 21 indicate the presence of clinically significant depression or anxiety. The authors of the study also translated the HADS into Arabic and found it to have good internal consistency, as indicated by a Cronbach’s alpha value of 0.87.

### Tool IV: Patient version of Person-centered Critical Care Nursing Questionnaire (PCCNPq)

The PCCNPq (Person-Centered Critical Care Nursing Perception Questionnaire) is a 20-item questionnaire developed by Hong and Kang (2020) to assess person-centered critical care nursing from the perspective of patients [39]. The questionnaire consists of five factors: compassion, expertise, communication, comfort, and respect. Each item is rated on a 4-point Likert-type scale, with response options ranging from 1 (strongly disagree) to 4 (strongly agree). Higher scores indicate a greater perception of individualized care. Concerning reliability, the questionnaire had acceptable internal consistency as Cronbach’s α of 0.89 to 0.91 [39] and had 0.91, in the present study.

### Statistical analysis

Data were fed to the computer and analyzed using IBM SPSS software package version 23.0. A one-way ANOVA test was used to compare more than two categories. Student t-test was used to compare two categories for normally distributed quantitative variables. Pearson coefficient was used to correlate between normally distributed quantitative variables. Linear regression was assessed to detect factors that affect **HADS** and **Kinesiophobia.** Path analysis was assessed using AMOS 23. 0 software to detect the Direct and Indirect Effect **of Person-Centered Critical Care Nursing on Kinesiophobia mediating by (HADS). s**ignificance of the obtained results was judged at the 5% level.

### Result

Concerning participant characteristics, 68.3% were female and 80 patients aged between 40 and 50 years old accounted for the largest proportion (30.2%) in Table 1. Regarding the level of education, illiterate was the largest proportion in the education level (*n* = 114,43%). More than half (52.5%) of the studied patients had working categorical was craft. Participants had congestive heart failure and rheumatic heart disease (23.0%, and 26.0% respectively), less than half of them (46.4%) experienced cardiac disease from 5 to 10 years, and 88.3% of them reported no other disease history. A statistical significance relation was found between all demographic characteristics and the HAD score. Moreover, age, gender, level of education, family history, diagnosis, and onset of disease were significantly correlated with the total score of the PCCNP questionnaire. Also, a statistical correlation was found between the Tampa Scale for Kinesiophobia, and demographic data including age (*p* = 0.024), sex (0.034), diagnosis (0.054), and level of education (0.047) **(see** Table 1**)**.

**Table 1:** A Descriptive Analysis of Demographics, Variation, and Correlations with HADS, PCCNPq, and TSK (*n* = 265)

Characteristic	Category	No. (%)	Anxiety		Depression		HADS*		PCCNPq*		TSK*	
			M (SD)	t/F (P)	M (SD)	t/F (P)	M (SD)	t/F (P)	M (SD)	t/F (P)	M (SD)	t/F (P)
**Gender**	Male	**84(31.7%)**	13.15 (4.97)	4.734* (< 0.001*)	13.83 (5.00)	6.742* (< 0.001*)	26.99 (9.89)	**5.737** ^*^ **(< 0.001** ^*^ **)**	39.37 (12.54)	**3.728** ^*^ **(< 0.001** ^*^ **)**	50.94 (9.47)	3.429* (0.024*)
	Female	**181(68.3%)**	9.94 (5.23)		9.64 (4.58)		19.57 (9.74)		45.91 (14.75)		50.35 (11.20)	
**Age**	< 20	**12(4.5%)**	0.00 (0.00)	18.493* (< 0.001*)	1.00 (0.00)	16.983* (< 0.001*)	1.00 (0.00)	**18.076** ^*^ **(< 0.001** ^*^ **)**	64.33 (2.46)	**7.114** ^*^ **(< 0.001** ^*^ **)**	56.42 (5.58)	3.418* (0.034*)
	20-<30	**45(17.0%)**	11.18 (6.57)		10.78 (6.10)		21.96 (12.63)		42.04 (16.63)		49.29 (11.61)	
	30-<40	**92(34.7%)**	12.47 (4.12)		12.49 (3.73)		24.96 (7.70)		43.57 (12.87)		49.37 (11.68)	
	40-<50	**80(30.2%)**	11.08 (4.10)		11.09 (4.44)		22.16 (8.46)		42.96 (13.24)		51.08 (10.09)	
	50-<60	**36(13.6%)**	10.22 (5.63)		10.36 (5.29)		20.58 (10.85)		41.86 (15.24)		51.94 (8.64)	
**Marital status**	Single	**68(25.7%)**	9.91 (7.17)	3.079* (0.028*)	9.62 (6.64)	4.864* (0.003*)	19.53 (13.78)	**3.927** ^*^ **(0.009** ^*^ **)**	43.66 (16.37)	**1.562 (0.199)**	49.79 (11.12)	**1.271 (0.285)**
	Married	**126(47.5%)**	11.99 (4.80)		12.17 (4.74)		24.16 (9.40)		42.28 (13.92)		49.75 (10.93)	
	Widowed	**11(4.2%)**	10.09 (3.62)		10.91 (3.02)		21.00 (6.63)		44.82 (14.57)		53.0(12.11)	
	Divorced	**60(22.6%)**	10.13 (3.78)		9.98 (3.28)		20.12 (7.03)		47.12 (12.63)		52.58 (9.15)	
**Education**	Illiterate	**114(43.0%)**	11.51 (4.70)	23.209* (< 0.001*)	11.68 (4.54)	22.007* (< 0.001*)	23.19 (9.15)	23.058* (< 0.001*)	40.67 (14.01)	**5.594** ^*^ **(< 0.001** ^*^ **)**	49.91 (11.26)	2.046* (0.047*)
	Primary school	**10(3.8%)**	9.00 (0.00)		10.00 (0.00)		19.00 (0.00)		47.00 (13.33)		53.20 (12.74)	
	Preparatory school	**17(6.4%)**	7.47 (0.87)		7.18 (0.53)		14.65 (0.93)		47.53 (14.16)		54.06 (6.69)	
	Secondary school	**28(10.6%)**	3.79 (4.07)		4.21 (3.46)		8.00 (7.52)		55.46 (13.46)		55.39 (5.73)	
	2 years institute after high school	**14(5.3%)**	16.00 (2.08)		15.50 (2.38)		31.50 (4.43)		41.86 (11.09)		48.50 (13.13)	
	University	**82(30.9%)**	12.74 (5.22)		12.40 (5.08)		25.15 (10.16)		43.45 (14.01)		49.05 (10.65)	
**Work**	Does not work/ student	**38(14.3%)**	9.63 (8.82)	4.770* (0.003*)	9.89 (7.92)	5.139* (0.002*)	19.53 (16.74)	4.995* (0.002*)	44.82 (18.71)	**2.417 (0.067)**	48.58 (12.15)	**0.607 (0.611)**
	Craft	**139(52.5%)**	12.04 (4.68)		12.08 (4.93)		24.12 (9.52)		41.73 (13.52)		50.63 (10.78)	
	Professional	**27(10.2%)**	10.93 (4.01)		10.37 (3.56)		21.30 (7.57)		48.19 (12.53)		50.62 (10.66)	
	Housewife	**61(23.0%)**	9.33 (3.74)		9.36 (2.81)		18.69 (6.40)		46.10 (13.50)		51.54 (9.73)	
**Diagnosis**	AF	**33(12.5%)**	13.24 (4.52)	18.735* (< 0.001*)	13.21 (5.82)	17.048* (< 0.001*)	26.45 (10.28)	18.042* (< 0.001*)	39.94 (10.43)	**3.979** ^*^ **(< 0.001** ^*^ **)**	50.21 (9.11)	2.204* (0.054)
	Aortic aneurysm	**16(6.0%)**	8.00 (1.03)		7.50 (0.52)		15.50 (1.55)		48.81 (13.58)		51.25 (11.15)	
	Cardiomegaly	**6(2.3%)**	7.00 (0.00)		9.00 (0.00)		16.00 (0.00)		44.67 (14.22)		51.17 (2.40)	
	CHF	**61(23.0%)**	15.03 (4.68)		14.82 (4.83)		29.85 (9.43)		40.67 (14.66)		48.34 (11.76)	
	HF	**34(12.8%)**	8.97 (2.50)		9.32 (2.03)		18.29 (4.46)		43.56 (14.06)		50.82 (11.92)	
	MI	**19(7.2%)**	7.05 (0.23)		9.00 (0.00)		16.05 (0.23)		40.68 (15.21)		50.21 (12.16)	
	RHD	**69(26.0%)**	8.68 (5.58)		8.35 (4.48)		17.03 (10.03)		50.13 (13.80)		52.42 (10.01)	
	SVT	**10(3.8%)**	7.00 (0.00)		7.00 (0.00)		14.00 (0.00)		47.00 (13.61)		54.60 (6.64)	
	UNSTEMI	**17(6.4%)**	16.00 (5.36)		15.18 (5.51)		31.18 (10.83)		34.41 (14.11)		47.94 (10.62)	
**Income**	Not enough	**136(51.3%)**	10.42 (5.76)	1.699 (0.091)	10.25 (4.98)	2.368* (0.019*)	20.67 (10.70)	2.037 (0.043*)	48.31 (13.76)	**5.475** ^*^ **(< 0.001** ^*^ **)**	51.01 (10.67)	**0.732 (0.961)**
	Enough	**129(48.7%)**	11.53 (4.83)		11.72 (5.13)		23.25 (9.86)		39.12 (13.56)		50.05 (10.69)	
**Onset**	< 5	**98(37.0%)**	10.36 (3.85)	7.598* (0.001*)	10.38 (4.08)	10.708* (< 0.001*)	20.73 (7.84)	9.188* (< 0.001*)	45.99 (12.07)	6.159* (0.002*)	51.34 (9.65)	**1.561 (0.185)**
	5-<10	**123(46.4%)**	12.19 (6.48)		12.31 (6.07)		24.50 (12.48)		40.64 (15.77)		48.88 (12.17)	
	10-<15	**44(16.6%)**	8.86 (3.66)		8.52 (2.34)		17.39 (5.97)		47.95 (13.43)		53.41 (7.16)	
**Are there any other diseases**	Yes	**31(11.7%)**	7.65 (0.95)	9.377* (< 0.001*)	8.32 (1.25)	7.244* (< 0.001*)	15.97 (2.17)	8.392* (< 0.001*)	46.55 (13.92)	**1.118 (0.265)**	53.87 (9.49)	**0.916 (0.503)**
	No	**234(88.3%)**	11.40 (5.54)		11.32 (5.31)		22.71 (10.76)		43.47 (14.44)		50.10 (10.76)	
**Family history**	Yes	**56(21.1%)**	7.55 (4.94)	5.771* (< 0.001*)	7.50 (4.31)	6.577* (< 0.001*)	15.05 (9.22)	6.182* (< 0.001*)	51.32 (13.00)	**4.544** ^*^ **(< 0.001** ^*^ **)**	50.70 (11.60)	**0.124 (0.199)**
	No	**209(78.9%)**	11.87 (5.09)		11.89 (4.90)		23.77 (9.88)		41.83 (14.11)		50.50 (10.43)	
**If yes.** (*n* = 56)	hypertension mother	**16(6.0%)**	12.25 (3.13)	42.856* (< 0.001*)	11.50 (2.68)	56.007* (< 0.001*)	23.75 (5.81)	48.185* (< 0.001*)	50.44 (10.11)	5.145* (0.003*)	44.56 (13.04)	**0.442 (0.587)**
	CHF father	**19(7.2%)**	2.11 (3.62)		2.58 (2.71)		4.68 (6.33)		59.16 (10.26)		55.84 (5.79)	
	Aortic aneurysm grandpa	**11(4.2%)**	8.82 (0.60)		7.91 (0.30)		16.73 (0.90)		43.00 (14.64)		48.45 (12.52)	
	Diabetes mother and father	**10(3.8%)**	9.00 (0.00)		10.00 (0.00)		19.00 (0.00)		47.00 (13.33)		53.20 (12.74)	

F: One-way ANOVA test t: Student t-test *: Statistically significant at *p* ≤ 0.05 * PCCNPq: Person-Centered Critical Care Nursing Questionnaire. * HADS: Hospital Anxiety and Depression Scale.*TSK: Tampa Scale for Kinesiophobia

Two models were generated to explore the relationship between the studied variables. Model 1 denotes the effect of PCCNP q on HADS. Model 2 represents the effect of PCCNPq on the TSK. Being female (B=-9.149, Beta= -0.412, t=-6.993, *p* < 0.001), and having enough income (B=-3.383, Beta= -0.163, t=-2.884, *p* = 0.004) were negatively associated with greater feelings of anxiety and depression in the studied cardiac patients. While statistically significance positive associated found with being married (B = 1.210, Beta = 0.125, t = 2.223, *p* = 0.027), onset (B = 0.585, Beta = 0.198, t = 3.474, *p* = 0.001), presence of additionally diseases (B = 12.491, Beta = 0.388, t = 6.098, *p* = 0.001) and Family history (B = 4.068, Beta = 0.161, t = 3.234, *p* = 0.001). To validate the relationship between the study variables, a regression analysis was performed, with the HAD scale as the mediator variable, PCCNP as the independent variable, and Kinesiophobia as the dependent variable. Model 1 shows that there is a moderate negative correlation (B=-0.295, Beta= -0.409, t=-8.061, *p* < 0.001) between the PCCNP questionnaire on HAD (R2 = 0.470, Adjusted R2 = 0.449, F = 22.512, *p* < 0.001). This means that the majority of being caring toward cardiac patients, the minor the feeling of anxiety and depression. Model 2 illumines that there is a high positive correlation (B = 0.377, Beta = 0.366, t = 0.762, *p* = 0.447) between the PCCNP questionnaire on Kinesiophobia (R2 = 0.080, Adjusted R2 = 0.073, F = 11.375, *p* < 0.001). In addition to being statistically significant, the beta coefficient for the Model2 effect of PCCNP q in the model is -0.295. This indicates that a lower Tampa Scale for Kinesiophobia score is linked to a stronger Model2 effect of PCCNP. Regression analysis results indicate that PCCNP is associated with decreased anxiety, despair, and mobility fear, suggesting that it is a helpful intervention for ICU cardiac patients **(see** Table 2**).**

**Table 2:** Linear Regression Analysis of Patient Characteristics and Effect of PCCNP q on HADS and TSK (*n* = 265)

		Model 1 Effect of PCCNP q on HADS				
	B	Beta	t	p	95% CI	
					**LL**	**UL**
Gender / Female	-9.149	-0.412	-6.993*	< 0.001*	-11.725	-6.572
Age	-0.105	-0.109	-1.695	0.091	-0.228	0.017
Education	0.028	0.006	0.105	0.916	-0.501	0.558
Marital status / Married	1.210	0.125	2.223*	0.027*	0.138	2.283
Work/working	-0.217	-0.021	-0.361	0.718	-1.398	0.965
Income / Enough	-3.383	-0.163	-2.884*	0.004*	-5.694	-1.073
Onset	0.585	0.198	3.474*	0.001*	0.253	0.916
Are there any other diseases /	12.491	0.388	6.098*	< 0.001*	8.457	16.525
Family history	4.068	0.161	3.234*	0.001*	1.591	6.545
PCCNP q*	-0.295	-0.409	-8.061*	< 0.001*	-0.367	-0.223
R^2^ = 0.470, Adjusted R^2^ = 0.449, F = 22.512^*^, *p* < 0.001^*^						
		**Model2 Effect of PCCNP q on TSK**				
	B	Beta	t	p	95% CI	
					**LL**	**UL**
HADS*	-1.280	-0.642	-1.338	0.182	-3.165	0.604
PCCNP q*	0.377	0.366	0.762	0.447	-0.596	1.349
R^2^ = 0.080, Adjusted R^2^ = 0.073, F = 11.375^*^, *p* < 0.001^*^						

F,p: f and *p* values for the model R^2^: Coefficient of determination B: Unstandardized Coefficients

Beta: Standardized Coefficients t: t-test of significance LL: Lower limit - UL: Upper Limit *: Statistically significant at *p* ≤ 0.05 * PCCNPq: Person-Centered Critical Care Nursing Questionnaire. * HADS: Hospital Anxiety and Depression Scale. *TSK: Tampa Scale for Kinesiophobia

Table 3 illustrates the correlation the relationship between anxiety, depression, nurse-patient care, and fear of movement in cardiac patients. The mean scores of HADS, PCCNP q, and TSK of 265 cardiac patients were 21.92± (10.36), 43.83± (14.39), and 50.54± (10.67), respectively. HADS had a strong correlation with the PCCNP q (*r* = 0.968), and the TSK (*r* = 0.992). This suggests that cardiac patients with higher anxiety and depression scores were also more likely to report symptoms of kinesiophobia and to experience PCCNP deficits. Pearson’s correlation analysis exposed that HADS were significantly negatively correlated between the scale PCCNP (r:-0.510, *p* < 0.001) while significantly positively correlated Tampa Scale for Kinesiophobia (r: 0.271, *p* < 0.001) correspondingly. Also, a significant positive correlation between the PCCNP questionnaire and the Tampa Scale for Kinesiophobia was found (*r* = 0.154, *p* = 0.012) **(see** Table 3**).**

**Table 3:** Correlation matrix of the relationship between HADS, PCCNPq, and TSK.

	Mean (SD)		Depression	Anxiety	HADS*	PCCNPq*	TSK*
Depression	10.96 (5.35)	**r**					
		**p**					
Anxiety	10.96 (5.10)	**r**	0.968^*^				
		**p**	< 0.001^*^				
HADS*	**21.92 (10.36)**	**r**	0.992^*^	0.992^*^			
		**p**	< 0.001^*^	< 0.001^*^			
PCCNPq*	**43.83 (14.39)**	**r**	-0.495^*^	-0.517^*^	-0.510^*^		
		**p**	< 0.001^*^	< 0.001^*^	< 0.001^*^		
TSK*	**50.54 (10.67)**	**r**	-0.279^*^	-0.259^*^	0.271^*^	0.154^*^	
		**p**	< 0.001^*^	< 0.001^*^	< 0.001^*^	0.012^*^	

r: Pearson Correlation. *: Statistically significant at *p* ≤ 0.05. * PCCNPq: Person-Centered Critical Care Nursing Questionnaire. * HADS: Hospital Anxiety and Depression Scale.*TSK: Tampa Scale for Kinesiophobia

**Fig. 2:**
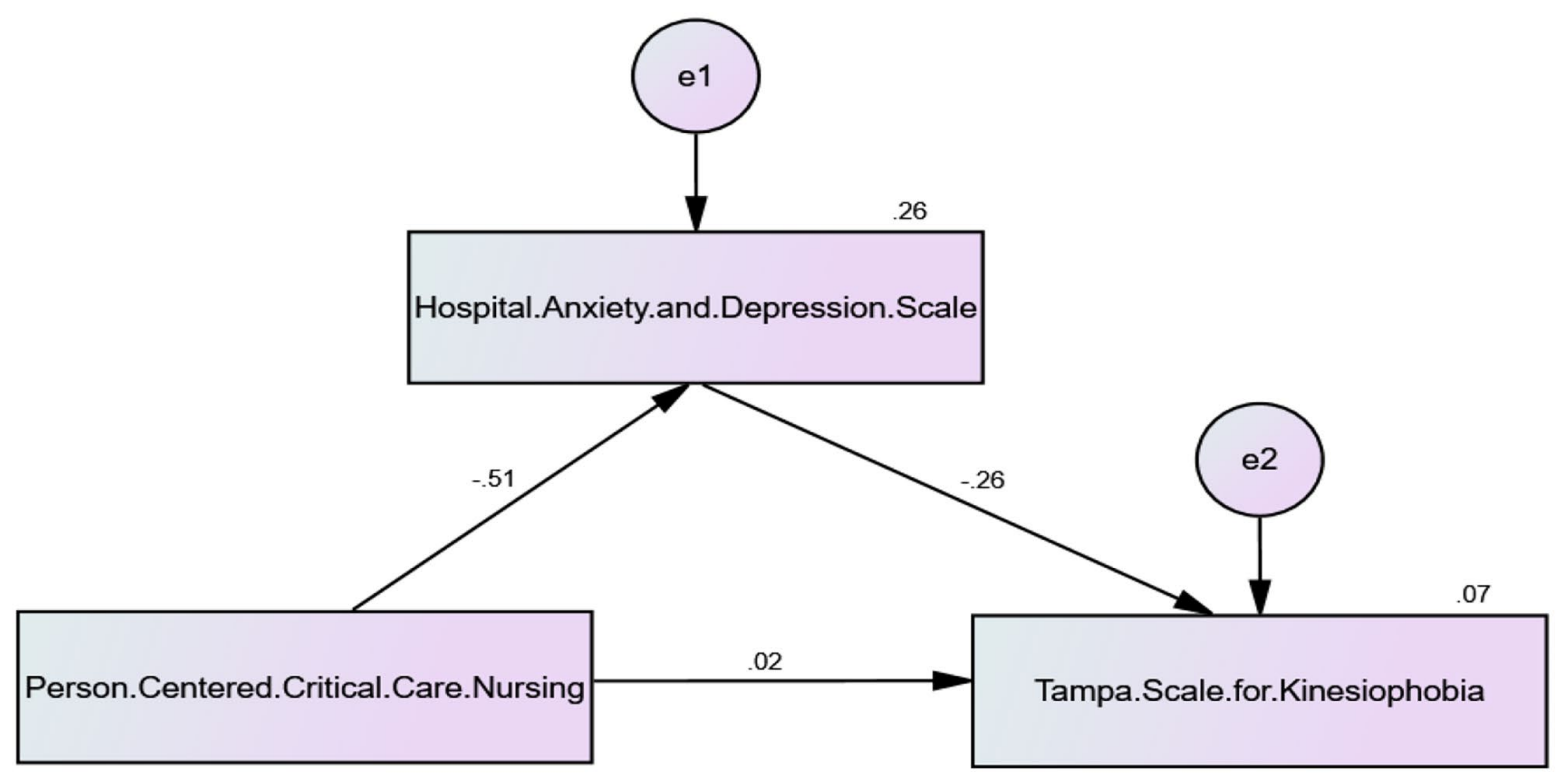
Path analysis to detect the Direct and Indirect Effect of Person-Centered Critical Care Nursing on Kinesiophobia mediating by Hospital Anxiety and Depression Scale (HADS). Model fit parameters CFI; IFI; RMSEA (1.000; 1.000; 0.350). CFI = Comparative fit index; IFI = incremental fit index; and RMSEA = Root Mean Square Error of Approximation. Model χ^2^; significance 33.248^*^(< 0.001^*^)

The study assessed the mediating role of HAD in the relationship between nursing care and kinesiophobia (**see** Table 4**&** Fig. 2). The results revealed a statistically significant direct effect (-0.367, CR= -9.628, *p* < 0.001)) of the effect of PCCNP on HADS. This means that cardiac patients have less anxiety and depression when receiving Person-Centered Critical Care Nursing. Furthermore, the direct effect of Person-Centered Critical Care Nursing (-0.015, CR = 0.302, *p* = 0.763) on Kinesiophobia in the presence of the mediator was also found to be not statistically significant. This indicates that there is no association between cardiac patients’ kinesiophobia and PCCNP upon ICU admission. Nonetheless, PCCNP indirectly affects kinesiophobia through HADS. With an indirect effect of -0.099, statistical significance is achieved. This indicates that by lowering anxiety and despair, PCCNP helps cardiac patients who are afraid to move. Hence, HADS partially mediated the relationship between Person-Centered Critical Care Nursing and Kinesiophobia. This means that in the model (Fig. 2), there is a significant negative correlation of 33.248 (< 0.001) in Path analysis.

**Table 4:** Direct and Indirect Effect of PCCNPq on TSK. mediating by HADS

Variable 1		Variable 2	Direct effect	Indirect effect	C.R	*p*-value
HADS	←	**Person-Centered Critical Care Nursing**	-0.367	0.0	-9.628^*^	< 0.001^*^
Kinesiophobia	←	**Person-Centered Critical Care Nursing**	0.015	-0.099	0.302	0.763
Kinesiophobia	←	**HADS**	-0.268	0.0	-3.787^*^	< 0.001^*^

## Discussion

The overarching aim of the current study was to examine the intricate associations between cardiac patients’ perceptions of nursing care and variables such as Kinesiophobia, depression, and anxiety. The empirical findings unveiled both direct and indirect impacts of person-centered critical care nursing on kinesiophobia. The mediation role played by anxiety and depression in this relationship provides a nuanced understanding of the multifaceted dynamics influencing patient outcomes within critical care settings. The direct effect implies that the implementation of person-centered care practices independently contributes to the amelioration of kinesiophobia among cardiac patients. This discernment underscores the intrinsic value of personalized and empathetic approaches inherent in person-centered care, engendering a heightened sense of control and comprehension for patients.

Bäck et al. emphasized that cardiac patients exhibit high levels of kinesiophobia, with a prevalence rate of 20% [9]. However, there is a lack of studies investigating kinesiophobia specifically in Egypt.

The indirect effect, mediated by anxiety and depression, underscores the intricate interplay between psychological factors and kinesiophobia in the context of critical nursing care. Anxiety and depression can heighten the perception of the threat associated with physical activity, leading to an exaggerated fear of movement or re-injury. These psychological states can impair coping mechanisms, reducing patients’ ability to manage and tolerate discomfort or perceived risk during physical activity, further reinforcing kinesiophobia. Additionally, anxiety and depression can contribute to a negative cycle of avoidance behavior, where patients withdraw from physical activities that they perceive as threatening, leading to deconditioning and increased kinesiophobia [40].

The correlation between the PCCNQ and the Kinesiophobia, suggests that patients who experience person-centered critical care nursing deficits are also more likely to report symptoms of kinesiophobia. This makes sense, as person-centered critical care nursing is designed to promote patients’ autonomy, control, and decision. Nursing care plays a crucial role in addressing these conditions, and significantly impacts the effectiveness of interventions. Patients who do not feel supported or understood by their caregivers may develop a sense of mistrust or fear, leading to increased anxiety about engaging in physical activities that could exacerbate their condition. Feeling neglected or misunderstood by healthcare providers may lead to a sense of vulnerability or lack of control, contributing to fear of movement. Furthermore, patients who perceive deficits in person-centered care may also be more likely to experience higher levels of overall distress, which can manifest as kinesiophobia [22].

The perception of nursing care among cardiac patients can significantly influence their levels of anxiety, depression, and subsequently, their experience of kinesiophobia. A positive perception of nursing care, characterized by empathy, attentiveness, and effective communication, can help alleviate anxiety and depression by fostering a sense of security and support. Patients who feel well-cared for may be more likely to engage in physical activities without excessive fear, reducing kinesiophobia. Conversely, a negative perception of nursing care, marked by perceived neglect, inadequate communication, or lack of support, can contribute to heightened anxiety and depression levels among patients. This negative experience may reinforce kinesiophobia as patients may feel less confident in their ability to safely engage in physical activities. Therefore, the perception of nursing care plays a crucial role in shaping the psychological well-being of cardiac patients and their ability to overcome kinesiophobia [41, 42]. This result emphasizes the interconnectedness of physical and psychological well-being, suggesting that improvements in mental health may play a pivotal role in alleviating kinesiophobia.

Consistent with this result, the investigation conducted by Bastani, et al. in 2022 [32], suggests that streamlining the admission and hospitalization processes for elderly patients in age-friendly medical facilities could potentially lead to a reduction in stress, anxiety, and depression among this demographic. Notably, hospitals with a clinical emphasis demonstrated high scores in care quality, corresponding to lower scores in anxiety and depression.

Furthermore, in their 2021 study, Westas and colleagues [27] found that patients with cardiovascular disease (CVD) often felt neglected in terms of their psychological needs, with healthcare professionals in cardiac care frequently overlooking depressive symptoms. The study emphasizes the importance of healthcare providers considering the overall well-being of CVD patients to identify and address depressive symptoms, fostering trust and preventing worsening health trajectories. Empowered CVD patients who can express their needs are more likely to receive assistance for depressive symptoms. To strengthen patient-provider relationships and support patients’ ability to address their needs, healthcare professionals should actively discuss and assess depressive symptoms, encouraging patients to express emotional challenges.

The intricate relationship between anxiety and cardiac issues creates a cycle wherein patients may exhibit altered movement patterns and behaviors. Heightened hypervigilance stemming from anxiety can make individuals acutely aware of bodily sensations associated with their cardiac condition, leading to a reluctance to engage in physical activities. This avoidance may extend to situations or activities perceived as potential triggers for discomfort or cardiac events, resulting in a sedentary lifestyle that exacerbates physical deconditioning. Patients may perceive exercise as a potential stressor, amplifying their anxiety and reinforcing kinesiophobia. Addressing anxiety in cardiac patients is vital not only for their mental well-being but also for breaking the cycle of kinesiophobia. Negative interpretations of symptoms influenced by anxiety further discourage participation in exercise, impacting adherence to cardiac rehabilitation programs. Social and cognitive factors, such as catastrophic thinking and social isolation, contribute to the development of kinesiophobia [43]. This is consistent with the study conducted by Fan et al. [44]., who concluded that individuals with coronary heart disease who undergo a specialized nursing intervention see improvements in various aspects, such as decreased anxiety and depression, enhanced quality of life related to angina, and better physiological outcomes.

The physical symptoms associated with cardiac conditions, such as chest pain or shortness of breath, can further contribute to a fear of movement. Additionally, cardiac rehabilitation programs especially in the acute stage, while essential for recovery, may inadvertently reinforce kinesiophobia by pushing patients to confront physical activities that trigger anxiety or discomfort. The fear of pain, injury, or exacerbating their cardiac condition can create a psychological barrier, preventing cardiac patients from engaging in regular physical activity [7]. Additionally, the current cardiac patients who have serious cardiac illnesses such as aortic aneurysm, congestive heart failure, supraventricular tachycardia or any other disease have higher levels of kinesiophobia.

The pervasive feelings of sadness and fatigue linked to depression can reduce motivation to participate in physical activities. As depression sets in, patients may lose interest in sustaining an active lifestyle, resulting in a more sedentary way of living. This decreased physical activity can lead to restricted movement, as individuals may steer clear of regular tasks or exercises that are vital for maintaining cardiovascular health [45, 46]. The current participants revealed a higher rate of depression which is correlated positively with kinesiophobia.

Depression typically has detrimental effects on individuals, and there is no scientific basis to suggest that it positively influences fear of movement among cardiac patients. Depression, as a mental health condition, tends to exert negative impacts on various aspects of a person’s life, including physical health. In the specific context of cardiac patients, depression is associated with reduced motivation, physical symptoms such as fatigue, and cognitive impairments. These factors contribute to a heightened fear of movement among cardiac patients, as they may perceive exercise as challenging or uncomfortable [47]. Additionally, negative perceptions and beliefs about their abilities, coupled with social withdrawal, can further reinforce kinesiophobia. Inconsistent with this point, kinesiophobia is positively correlated with depression in the current study.

A cardiac diagnosis often brings about significant lifestyle changes, such as dietary restrictions, medication regimens, and the necessity for regular medical monitoring. These adjustments can lead to feelings of loss, frustration, and a sense of diminished control over one’s life, contributing to the development of depression. The physical symptoms associated with cardiac conditions, including fatigue and shortness of breath, can further exacerbate feelings of helplessness and despair [48]. The fear of mortality and the potential limitations on daily activities can instill a persistent sense of anxiety and sadness. Social isolation, common among cardiac patients due to lifestyle modifications or perceived fragility, can also contribute to the prevalence of depression [49].

Moreover, the physiological impact of cardiovascular issues on the brain, through mechanisms such as reduced blood flow or inflammation, can directly contribute to depressive symptoms. Dhar and Barton (2016) [50] concluded that the intricate relationship between Major Depressive Disorder (MDD) and Coronary Heart Disease (CHD) involves complex and multifactorial mechanisms, including the sympathetic nervous system, platelet hyperactivity, inflammation, and dysregulation of the hypothalamic-pituitary-adrenal (HPA) axis, among others. Conducting a definitive mortality study is challenging due to the complexities and costs associated. However, the current evidence underscores the importance of optimizing efficacy and minimizing potential harm when selecting treatments for individuals with MDD and comorbid CHD. It is suggested that MDD should be regarded as a common and modifiable risk factor for CHD, similar to established factors like smoking, hypertension, and hyperlipidemia. The detrimental combination of MDD and CHD results in adverse health outcomes for both conditions, contributing to escalating movement restrictions [11, 51, 52].

Men in the current study revealed higher kinesiophobia, depression and anxiety. Socialization norms that dictate traditional masculine roles may lead men to suppress emotions and resist seeking mental health support. Men with cardiac disorders also may experience higher levels of stress due to concerns about their health, financial burdens, or the impact of the condition on their ability to fulfil societal roles [53]. If they lack adaptive coping mechanisms or perceive seeking help as a sign of weakness, they may be more prone to developing symptoms of depression and anxiety. Moreover, cardiac disorders can lead to physical limitations and lifestyle changes, affecting an individual’s sense of identity, self-esteem, and independence [54]. For males who traditionally associate their self-worth with physical prowess and independence, these changes may be particularly challenging to navigate, contributing to feelings of depression and anxiety. The fear of exercise, particularly in the context of cardiac disorders, may further contribute to psychological challenges [41, 55].

Cardiac patients engaged in craft work who also experience financial constraints may exhibit heightened levels of kinesiophobia individuals with limited financial resources may face challenges accessing appropriate healthcare and rehabilitation services, hindering their ability to receive tailored guidance on safe and gradual physical activity [7, 56]. The fear of exacerbating their cardiac condition without proper supervision could intensify their aversion to movement. Furthermore, the economic strain itself may contribute to heightened stress and anxiety, as financial worries are known stressors [57]. This additional psychological burden can magnify concerns about the potential risks associated with physical exertion, reinforcing kinesiophobia. Moreover, engaging in craft work may involve prolonged periods of sedentary behavior, which can contribute to deconditioning and a heightened sense of vulnerability during physical activity [11]. The intersection of financial constraints, limited access to healthcare resources, and the sedentary nature of certain occupations can thus create a complex interplay that fosters kinesiophobia among cardiac patients involved in craft work with insufficient income.

## Conclusion

A notable correlation was observed between the perception of nursing care and kinesiophobia anxiety and depression in the cardiac participants. Patient’s demographic and clinical characteristics such as being female, married, having sufficient income, experiencing the onset of cardiac disease, having comorbid health conditions, and having a family history are associated with the reduced likelihood of heightened feelings of anxiety and depression among participants. The regression analysis revealed that the perception of nursing care is negatively linked to anxiety, depression, and mobility-related kinesiophobia among the studied rural cohorts.

### Implication

The study’s outcomes are of vital necessity in customizing an individualized cardiac rehabilitation program (CR) based on the emotional experience of cardiac patients, which will be conducive to rehabilitation and prognosis for patients, thereby lessening the physical burden and improving their quality of life. Additionally, it grants the interdisciplinary collaboration of the nursing staff, physicians, and psychologists to lay out psychoprophylactic programs and take precautions against kinesiophobia by reducing feelings of fear and anxiety linked to it among post-CVD patients. The existing findings also have implications for holistic nursing care in terms of early identification of barriers to physical activity, improved effectiveness of the recovery process, and averting recurrent hospital stays. Considering the relationship of kinesiophobia with mild to moderate physical activity, clinicians may have taken precautions against encouraging individuals with MI to engage in physical activity. Further studies should detail the relationship between physical activity and kinesiophobia in more comprehensive physical activity monitoring from MI patients with a pedometer or sensor-based devices.

## Data Availability

No datasets were generated or analysed during the current study.
